# Supplementary material for: Expert Opinions about Barriers and Facilitators to Physical Activity Participation in Ghanaian Adults with Type 2 Diabetes: A Qualitative Descriptive Study
Source: Sports (Basel). 2023 Jun 21;11(7):123. doi: 10.3390/sports11070123 (PMC10383987; doi:10.3390/sports11070123)
Supplement: Supplementary file 1 [file sports-11-00123-s001.zip › sports-2468649-supplementary.pdf]

Supplementary File S1. Semi-Structured Interview Guide for the Expert Group

Hello, my name is Mohammed Amin. I am a student researcher undertaking my PhD. The project is titled, 'Design of a Physical Activity Program to Improve Metabolic Syndrome in Ghanaian Adults with Type 2 Diabetes Mellitus'. Thank you for volunteering to take part in our study. I appreciate your time and commitment. Today I will be asking you a series of questions related to the design of physical activity programs for people with type 2 diabetes in Ghana. This interview will be recorded to ensure we capture your comments and will only be used for the purpose of this research. Did you understand what this study implies and do you consent to take part in it? Do you consent to your interview being recorded? Do you have any questions for me before we get started? If not, let's begin!

Section A: Personal and Professional Information

1. Age group:
   a) 18-35
   b) 36-55
   c) above 55 years

2. Gender:
   a) Male
   b) Female
   c) Prefer not to disclose

3. Profession: ________________________________

4. What is your area of expertise/practise ________________________________

5. How many years of experience do you have in your area of practise? ________________________________

6. How many years of working experience relative to your area of practise do you have in Ghana?

7. 7. What is your current affiliated institution? ______________________________

Section B: Physical Activity Behaviour

---

1. What are the barriers to physical activity participation among people with type 2 diabetes?
2. What can be done to support them to overcome those barriers?
3. What are some strategies to build confidence in people with T2DM towards achieving their physical activity targets?
4. How best can people with type 2 diabetes be supported to develop positive thoughts about physical activity?
5. How best can people with type 2 diabetes be supported to reduce their perceived physical activity barriers/challenges?
6. How best can people with type 2 diabetes be supported to incorporate physical activity into their daily routine?
7. How can they utilise activities of daily living to achieve physical activity targets?
